# MicroRNA Expression Signature in Mild Cognitive Impairment Due to Alzheimer’s Disease

**DOI:** 10.1007/s12035-020-02029-7

**Published:** 2020-07-31

**Authors:** Bruna De Felice, Concetta Montanino, Mariano Oliva, Simona Bonavita, Valeria Di Onofrio, Cinzia Coppola

**Affiliations:** 1 Department of Environmental, Biological and Pharmaceutical Sciences and Technologies (DISTABIF), University of Campania “Luigi Vanvitelli”, Via Vivaldi 43, 81100 Caserta, Italy; 2 Department of Advanced Medical and Surgical Sciences, University of Campania “L. Vanvitelli”, Naples, Italy; 3 Department of Sciences and Technologies, University of Naples “Parthenope”, Naples, Italy

**Keywords:** Mild cognitive impairment (MCI), microRNAs, Hsa-mir-567, Alzheimer’s disease (AD)

## Abstract

Mild cognitive impairment (MCI) defines an intermediate state between normal ageing and dementia, including Alzheimer’s disease (AD). Identification of MCI subjects who will progress to AD (MCI-AD) is today of crucial importance, especially in light of the possible development of new pathogenic therapies. Several evidences suggest that miRNAs could play relevant roles in the biogenesis of AD, and the links between selected miRNAs and specific pathogenic aspects have been partly explored. In this study, we analysed the composition of microRNA transcriptome in blood, serum and cerebrospinal fluid samples from MCI-AD subjects, from an enriched small RNA library. Real-time qPCR from MCI-AD and AD patients and normal controls was performed to profile miRNA expression. In particular, four microRNAs, hsa-mir-5588-5p, hsa-mir-3658, hsa-mir-567 and hsa-mir-3908, among all selected microRNAs, are dysregulated. Hsa-mir-567 was found to be differentially expressed in cerebrospinal fluid samples, blood and serum from MCI-AD patients, showing the highest fold change and statistical significance. Target prediction analysis have been performed to evaluate mRNAs whose expression was controlled by miRNAs found to be dysregulated here, showing that hsa-mir-567 target genes are functionally active in neuronal cells. We propose that miRNA profiles found in samples from MCI-AD patients might be relevant for a better understanding of AD-related cognitive decline and could lead to set up suitable and potential biomarkers for MCI-AD progression to AD.

## Introduction

Neurodegenerative diseases, such as Alzheimer’s disease (AD), are age-related disorders whose prevalence is growing worldwide due to the progressive ageing of the population, therefore constituting a very important public health problem. Mild cognitive impairment (MCI) is a condition characterized by a subjective experience of progressive decline of cognitive functions, accompanied by objective evidence of altered cognitive performance, without significant impairment of daily life activities [1]. MCI can be divided in two subtypes: an amnesic form, if the memory domain is impaired, and a non-amnesic form, if a cognitive domain different from memory is altered. Cognitive deficits can also affect more than one domain, and therefore MCI can be further classified in single domain or multiple domains. In some cases (from 12 to 20% per year) [2, 3], MCI patients progress to a dementia syndrome, while in other cases they remain stable or even regress. In recent years, specific criteria have been developed to identify the proportion of MCI whose underlying pathology is cerebral amyloidosis [4]. These “MCI due to AD” (MCI-AD) subjects should be considered to be in the pre-dementia phase of AD; therefore it is assumed that they will inevitably progress to fully developed AD over time. The classification of a MCI subject as “MCI-AD” requires the positivity of at least one biomarker of in vivo AD pathology, either CSF amyloid-β 1–42 (Aβ_42_), total tau (T-tau) and 181-phopshoprylated tau (P-tau) assay or amyloid tracer PET. Thus, molecular factors as microRNAs could be potential biomarkers of progression in MCI-AD. MicroRNAs (miRNAs) are small non-coding RNA molecules (sncRNA) that act as endogenous regulators of gene expression by binding to complementary sequences of target messenger RNAs (mRNAs) and subsequently inducing either translational repression or mRNA destabilization. MiRNAs play a key role in many neurobiological processes, since they are widely present within the nervous system, such as neurodevelopment, neuroplasticity, apoptosis and others [5]. Some miRNAs are highly expressed in specific neuronal compartments, including axons, dendrites and synapses [6, 7], where they are essential for normal neuronal function and survival [8]. Impaired expression of several miRNAs has been found in various neurodegenerative conditions, including AD, Parkinson’s disease and amyotrophic lateral sclerosis [9–12], as well as in non-neurodegenerative dementias such as vascular dementia [13]; however, only very few studies have specifically focused on the earliest stages of the disease. MiRNAs have been shown to be stable in blood samples [14], and measurements of altered miRNA expression patterns in blood, serum and CSF have become promising novel diagnostic tools and prognostic biomarkers for various neurological diseases [15].

MicroRNAs have been increasingly implicated in AD and other neurological diseases. MicroRNAs could be useful as potential biomarkers for progression of MCI to dementia [16]. The results from such studies showed microRNAs with a modest specificity, except for miR-206, which was differentially expressed in high level from serum of MCI patients converting to AD (no IWG-2 criteria were included) [17].

In our research, we selected four miRNAs, namely, hsa-mir-5588-5p, hsa-mir-3658, hsa-mir-567 and hsa-mir-3908, from an enriched small RNA library and analysed their expression in various biological samples (serum, blood, CSF) from a cohort of MCI-AD (according to the IWG-2 criteria) and AD-dementia patients. The selected miRNA expression was analysed for validation by qRT-PCR. Among such microRNAs, mir-567 was highly expressed showing a significant expression change between MCI-AD and AD patient groups in all biological samples. miR-567 showed the highest fold change and significance, having the potential role as not invasive and easily detected biomarker for MCI-AD pathology. Moreover, we performed a target prediction analysis intersecting the results from TargetScan, Pictar and mirBase, to evaluate mRNAs whose expression is controlled by miRNAs found to be dysregulated in our study. This research provides a starting point for future studies aimed at understanding the roles of these miRNAs in AD-related cognitive decline from the earliest stages of disease and their potential use as AD pathology biomarkers.

## Materials and Methods

### Ethics Statement

We obtained ethics approval for the study from our institution ethics committee (also known as an Institutional Review Board). All the participants had the capacity to consent and a written informed consent has been acquired from all of them.

### Subjects

We enrolled 18 MCI-AD and 18 AD patients (Table 1), whose diagnosis was reached according to the IWG-2 criteria [4]. A complete laboratory assessment was performed, including dosage of vitamin B_12_ and folic acid, study of thyroid function and serum diagnosis for syphilis. Extended neuropsychological evaluation allowed further classifying MCI-AD subjects in the various clinical subtypes outlined by Petersen and colleagues [18]. Investigations of structural and functional neuroimaging (brain MRI and ^18^FDG-PET) were carried out. In all patients, the diagnosis was supported by the positivity of at least one in vivo biomarker of AD neurodegeneration, either CSF biomarkers (27/36 patients) or amyloid tracer PET (9/36 patients). CSF biomarkers consisting of levels of T-tau, P-tau and Aβ_42_ were determined with human-specific ELISA kits (Innogenetics). A CSF profile consisting of decreased Aβ_42_ together with increased T-tau or P-tau concentrations was considered diagnostic for AD underlying pathology. Cut-off values for CSF Aβ_42_, T-tau and P-tau have been set to 500, 400 and 61 pg/ml, respectively. ^18^F-florbetapir was the radiotracer used for amyloid PET imaging. Visual assessment of the regional cortical tracer uptake in four different brain regions (lateral temporal, frontal, posterior cingulate/precuneus and parietal lobes) allowed classifying amyloid PET scan as “positive” or “negative”. In addition to cognitively impaired patients, we enrolled 30 age- and sex-matched healthy controls, in which any cognitive alteration was excluded by means of an extended neuropsychological evaluation.

**Table 1:** Demographic and clinical characteristics of MCI and AD patients

	MCI-AD group (*n* = 18)	AD group (*n* = 18)
Age at our observation (years)		
Mean	69.9 (± 5.04)	70.8 (± 10.22)
Range	60–76	51–85
Age at symptoms onset (years)		
Mean	67.5 (± 5.60)	67.2 (± 10.48)
Range	56–74	50–84
Sex		
Male	9 (50%)	6 (33%)
Female	9 (50%)	12 (67%)
Mean MMSE score at our observation	26.39 (± 2.03)	16.77 (± 4.87)
Clinical subtypes (for MCI patients)		
Amnesic single domain	3 (16.7%)	
Non-amnesic single domain	3 (16.7%)	
Amnesic multiple domain	9 (50.0%)	
Non-amnesic multiple domain	3 (16.7%)	

Data are presented as *n* (%) or mean ± SD

### RNA Extraction

Peripheral blood samples have been obtained from MCI-AD and AD patients. For RNA isolation and purification from whole blood, the Trizol (Invitrogen, #15596-026) method has been used for all samples within 1 h from drawing, thus reducing RNA degradation. The RNA isolated with this protocol comes from all white cells, including polymorphonuclear leukocytes and mononuclear cells. RNA was isolated including an optional DNase digestion step. This standardized RNA isolation procedure guarantees high-quality non-degraded RNA. RNA samples were quality-checked by the identification of 18S rRNA and 28S rRNA peaks via the Agilent 2100 Bioanalyzer platform (Agilent Technologies). Serum and CSF samples were also extracted from all MCI-AD and AD patients, centrifuged at 12,000 × g for 5 min at 4 °C and stored at − 80 °C for total RNA extraction using Trizol reagent (Invitrogen). Finally, with the same procedures outlined above, serum samples from healthy controls were extracted and processed.

### Small RNA Library Preparation and Sequencing

The construction of three small RNA libraries was carried out as previously described [19]. Total RNA (30 μg) was pooled in 3 pools, each comprising RNA from peripheral blood leukocytes of enrolled patients. RNA pools were size fractionated on 15% Tris/Borate/EDTA urea polyacrylamide electrophoresis gel, and the sncRNA fraction in the size range of 18 to 80 nt was extracted, purified and cloned. Roughly 1000 cloned sncRNA molecules were independently sequenced for each library. Colony PCR was performed using 5′ and 3′ primers, and the clones with PCR products about 110 bp in length were sequenced.

### Reverse Transcription-Quantitative Polymerase Chain Reaction

cDNA was obtained by using oligo-dT primers or stem-loop reverse transcriptase (RT) primers, respectively. RNU6B was used as controls for miRNAs. Real-time qPCR was performed under the following conditions: 94 °C for 4 min, followed by 40 cycles at 94 °C for 1 min, 56 °C for 1 min and 72 °C for 1 min. Relative expression levels of hsa-mir-5588-5p, hsa-mir-3658, hsa-mir-567 and hsa-mir-3908 were calculated using the 2^-ΔΔCt^ method.

### Statistics

The results are mean ± SD of at least three separate experiments, measuring each parameter by triplicate (*n* = 3). Statistical significant differences were tested by one-way analysis of variance (ANOVA), and, when the F value was significant, by Student-Newman-Keuls test. *P* value less than 0.05 (*) was considered statistically significant.

### MiRNA Target Prediction

Target mRNAs that have the potential binding sites for individual miRNAs were identified by searching them on public databases endowed with prediction algorithms, such as TargetScan (http://targetscan.org), PicTar (http://pictar.mdc-berlin.de), miRBase (http://www.mirbase.org), TarBase (http://microrna.gr/tarbase) and Miranda (http://microrna.sanger.ac.uk/sequences).

We considered those found to be significant in both methods potential relevant targets, as previously suggested [20]. String database was used to build the protein-protein interaction (PPI) network and to perform Gene Ontology and functional annotation [21].

## Results

### MiRNA Distribution in Libraries from MCI-AD Patient’s Blood

**Fig. 1:**
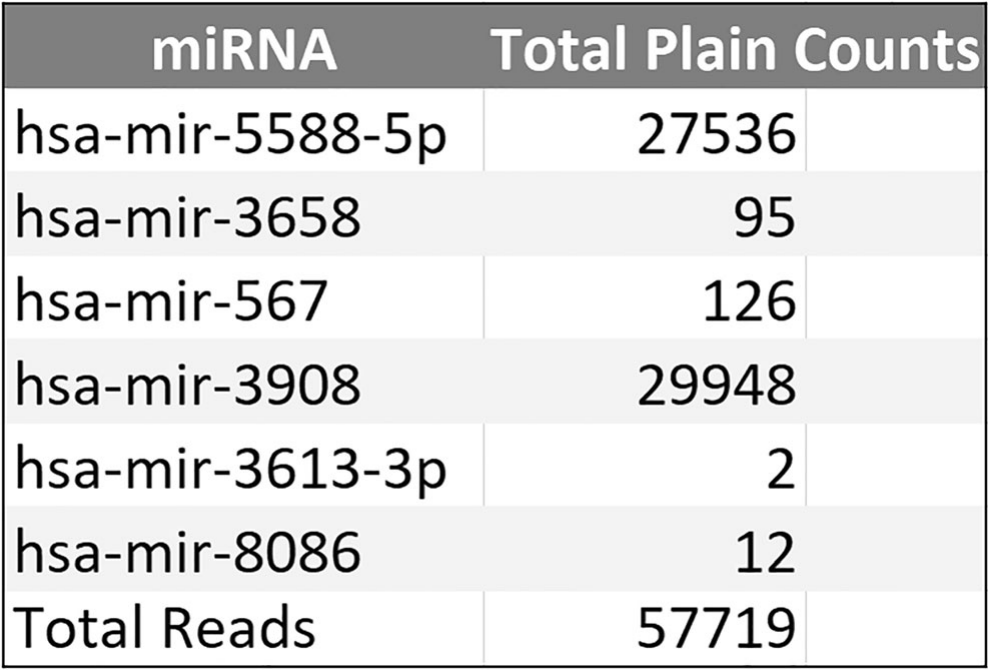
MicroRNAs identified by small RNA cloning from serum of MCI patients

According to the primary aim of characterizing the sncRNA signature from peripheral blood of MCI-AD cases, we evaluated a set of 18 MCI-AD patients by sncRNA cloning. About 60,000 cloned sncRNA molecules were independently sequenced for each library. This approach allowed us to obtain a profile of sncRNAs expressed in blood leukocytes. From these sncRNAs, we were able to isolate 6 mature miRNA species (Fig. 1), namely hsa-mir-5588-5p, hsa-mir-3658, hsa-mir-567, hsa-mir-3908, hsa-mir-3613-3p and hsa-mir-8086, deriving from known human stem-loop sequences (miRBase 12.0).

### Expression of Hsa-Mir-5588-5p, Hsa-Mir-3658, Hsa-Mir-567 and Hsa-Mir-3908 in MCI-AD and AD Patients and in Healthy Controls

**Fig. 2:**
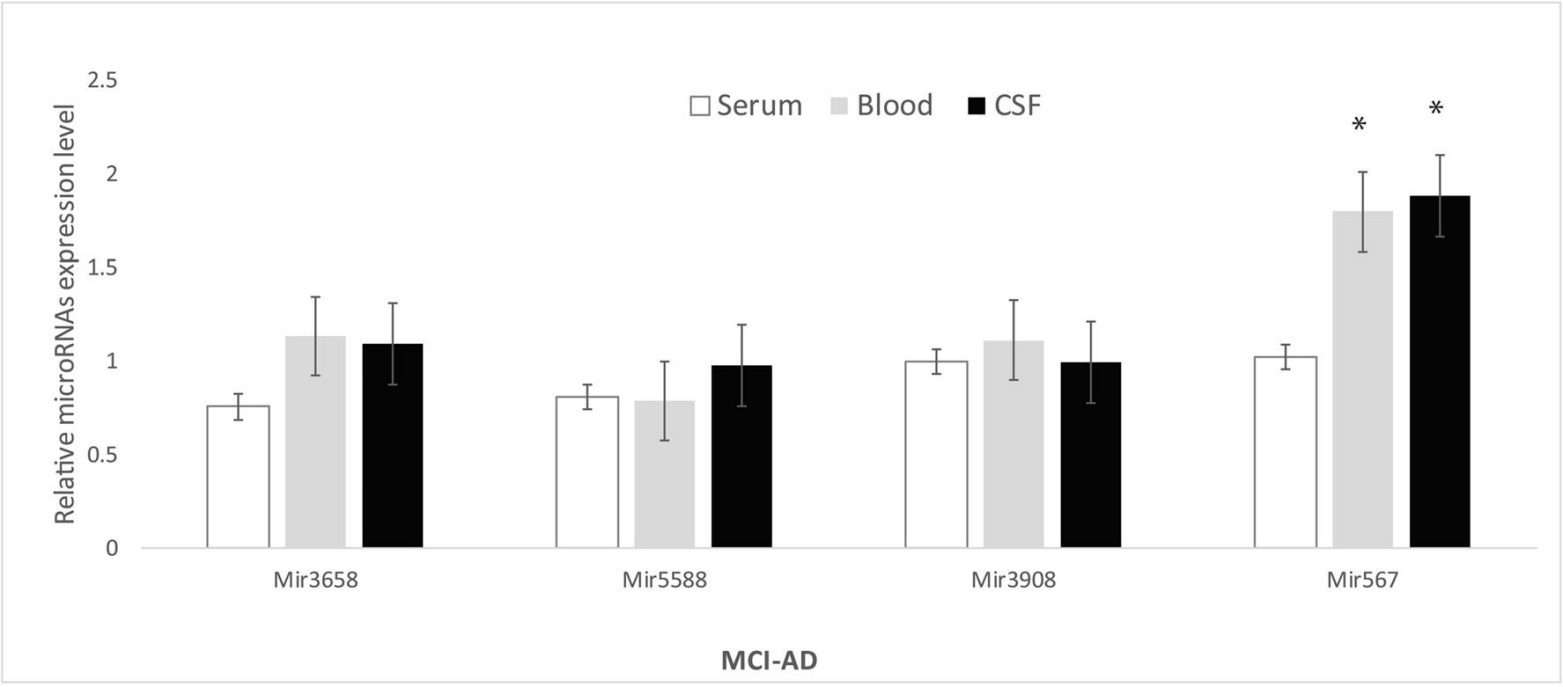
MicroRNA expression levels in the leukocytes of MCI-AD patients. The expression of microRNAs was studied in blood leukocytes, serum and CSF of MCI patients, by microRNA assay-based quantitative real-time PCR following the delta-delta Ct method. Statistically significant differences were tested at *p < 0.05

**Fig. 3:**
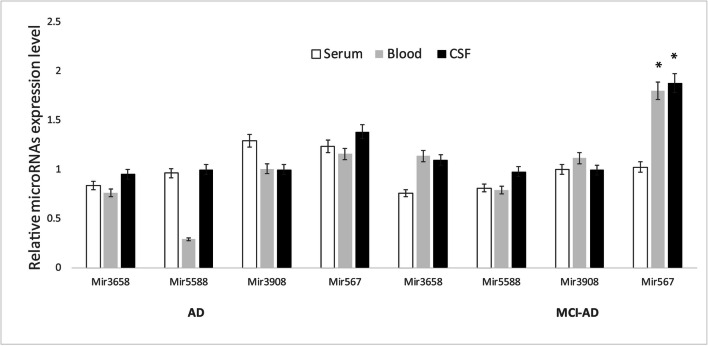
MicroRNA expression levels in the leukocytes of MCI-AD and AD patients. The expression of microRNAs was studied in blood leukocytes, serum and CSF from MCI and AD patients, by microRNA assay-based quantitative real-time PCR following the delta-delta Ct method. Statistically significant differences were tested at *p < 0.05

**Fig. 4:**
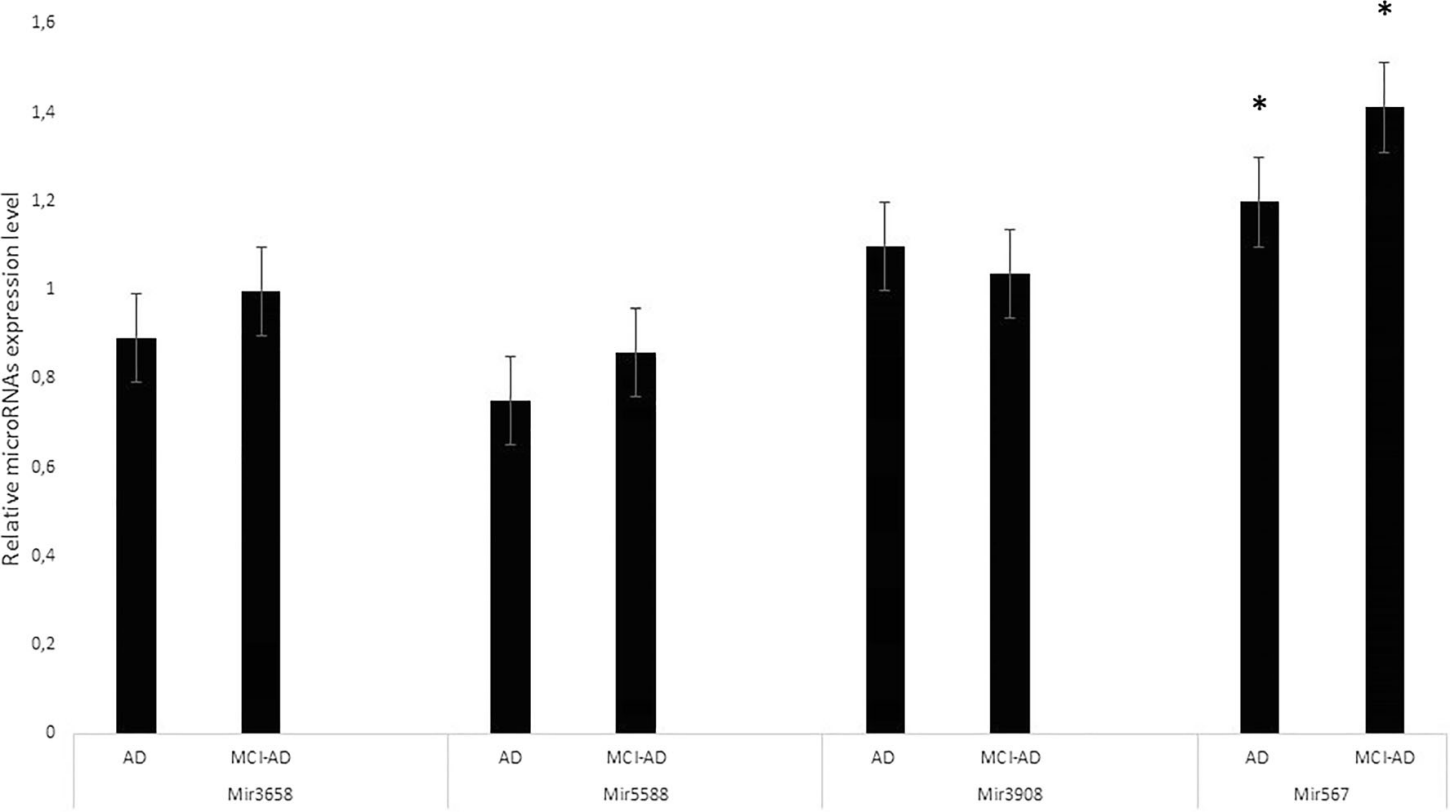
Average level of hsa-mir-5588-5p, hsa-mir-3658 and hsa-miR-567 expression from MCI-AD and AD patients. *p < 0.05

**Fig. 5:**
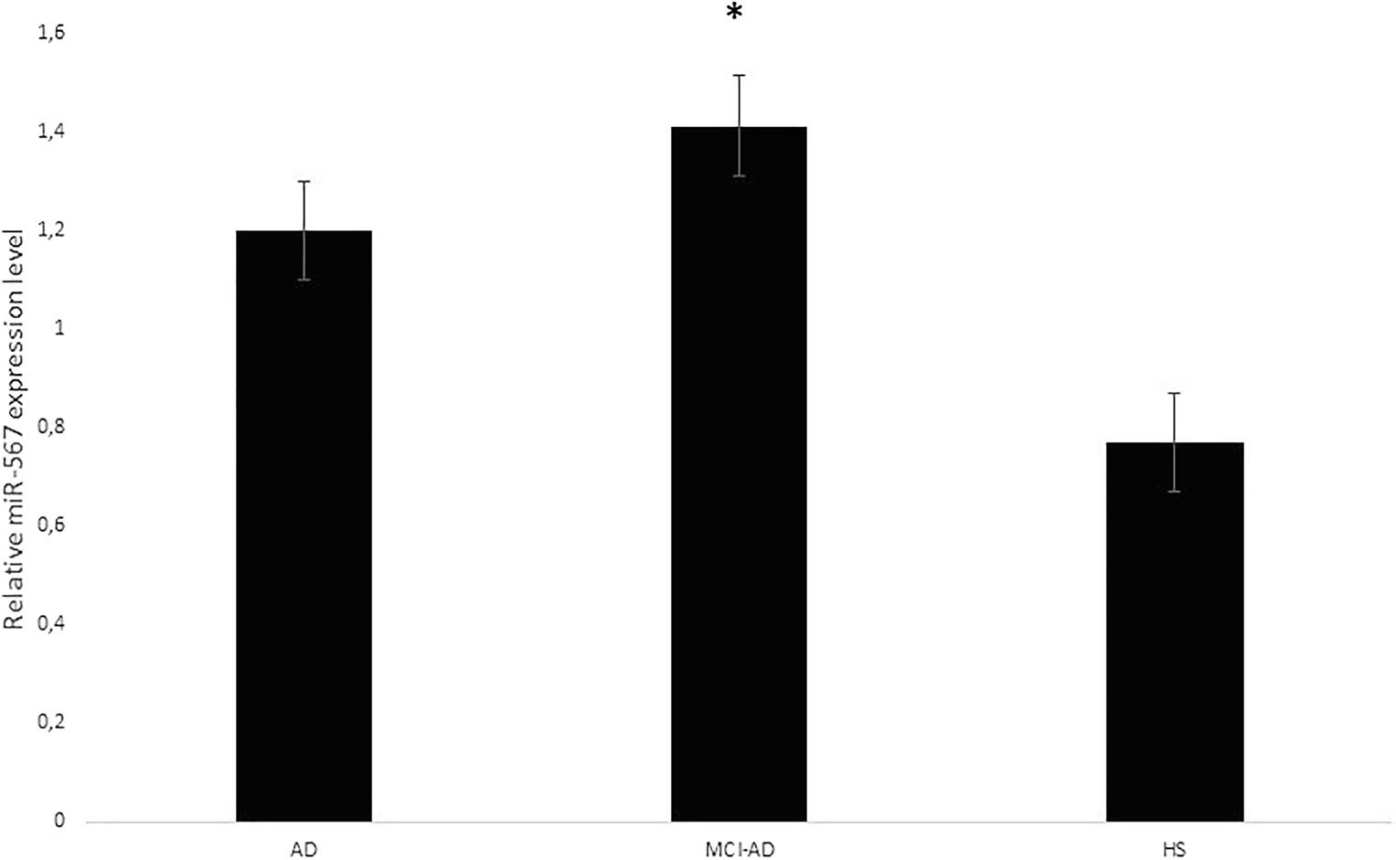
Real-time PCR analyses of hsa-miR-567 in sera from MCI-AD and AD patients versus healthy age-matched subjects (HS). *p < 0.05

To gain a quantitative profile of the expression, we selected the most abundant miRNAs, namely hsa-mir-5588-5p, hsa-mir-3658, hsa-mir-567 and hsa-mir-3908, to detect their expression in blood leukocytes as well as in CSF and serum from MCI-AD patients. Using real-time strategy, we detected that, among these, only hsa-miR-567 has been found to be highly expressed in blood, leukocytes and CSF, substantially upregulated with a twofold induction in the group of MCI-AD patients compared with the other selected miRNAs (p* < 0.05) (Fig. 2). Subsequently, we compared miRNA profiles of our MCI-AD patients with a group of 18 age- and sex-matched AD patients, in order to explore a possible variability in the miRNA expression profile between these two groups. We found that three of such four miRNAs were more expressed in MCI-AD compared with AD patients (p*-value cut-off < 0.05). Indeed, hsa-mir-5588-5p, hsa-mir-3658 and hsa-mir-567 were upregulated in blood leukocytes as well as in CSF and serum from MCI-AD compared with AD patients (Fig. 3), whereas hsa-mir-3908 was downregulated in MCI-AD. Among miRNAs that displayed a different expression profile between MCI-AD and AD groups, interestingly, hsa-mir-567 showed the highest fold changes and the highest statistical significance in MCI-AD in leukocytes, serum and CSF (p* < 0.05) (Fig. 4). CSF represents the most helpful biological sample for the evaluation of potential biomarkers of neurodegenerative diseases. In fact, since it is in close contact with the central nervous system, it could reflect biochemical and/or physiological changes that occur in the brain. However, miR-567 was upregulated in blood and serum too, indicating a common upregulated microRNA in all the tissues from MCI-AD patients. Moreover, in order to evaluate a potential role of hsa-mir-567 as AD pathology biomarker, we compared hsa-mir-567 serum expression in MCI-AD and AD patients with serum from 30 healthy age- and sex-matched subjects. This further analysis showed a significant upregulation of hsa-mir-567 in MCI-AD and AD patients compared with healthy controls (p* < 0.05) (Fig. 5). This result could be important and must be further verified, especially by correlating it with the progression of MCI-AD in AD. From this point of view, the miR-567 variations could represent a potential and early biomarker of progression of MCI-AD in AD.

### Computational Predictions of the Putative Targets of miR-567

The overlap between putative targets of miRNAs and the expression of mRNAs provides information on the biological functions and networks of genes regulated by specific miRNAs. Therefore, we explored putative hsa-mir-567 target genes by searching them on three distinct web-accessible miRNA target databases, including TargetScan, PicTar and miRDB. In particular, we submitted the target genes obtained from this approach to the KEGG pathway and Gene Ontology tools, both implemented in the String database.

**Fig. 6:**
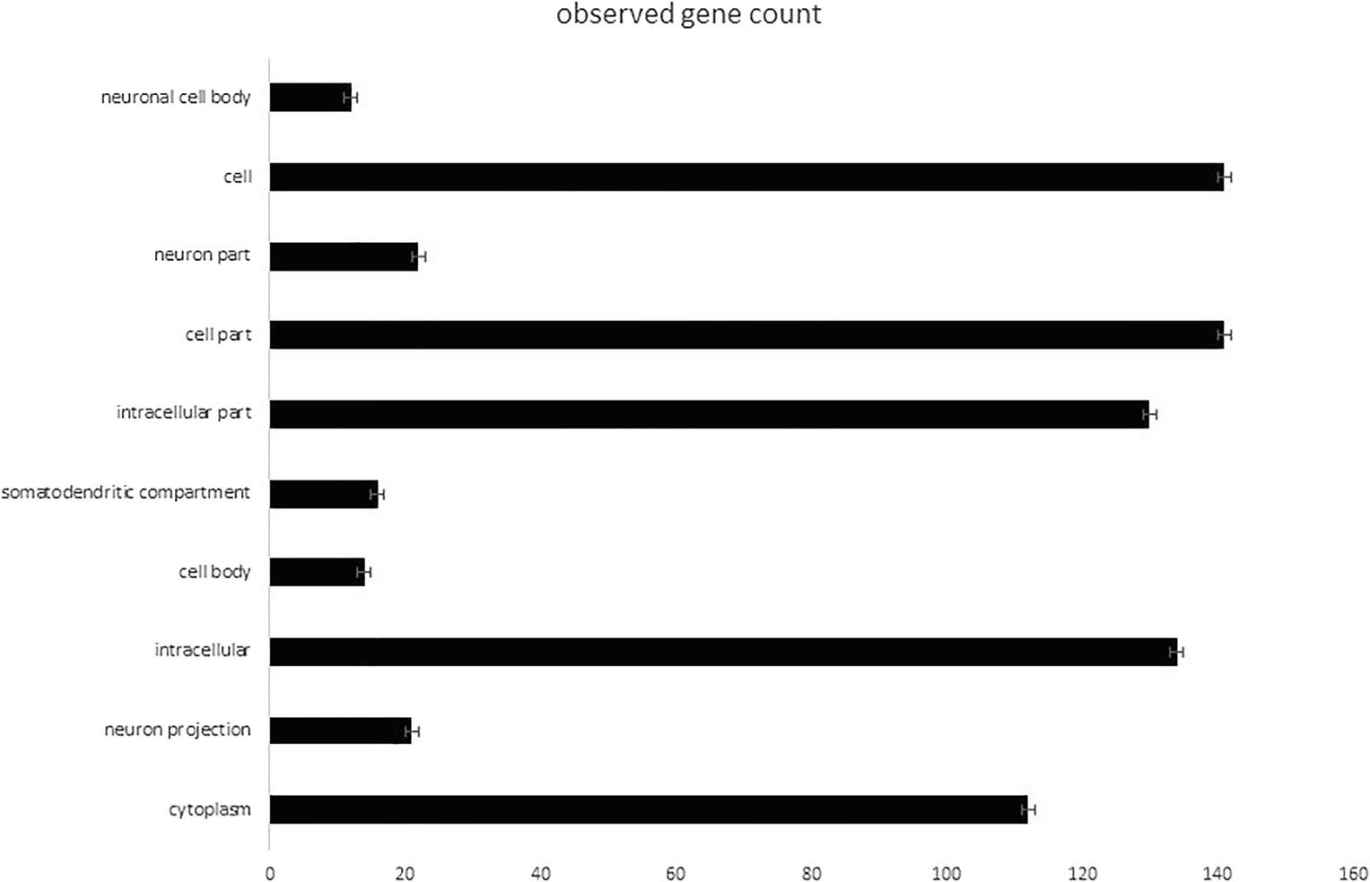
Bar plot showing the number of MCI-associated genes enriched in each of reported GO term

Regarding the Gene Ontology analysis, as shown in Fig. 6, a significant pathway enrichment was observed in the cellular component category indicating that hsa-mir-567 target genes are functionally active mainly in neuronal cells. We observed a statistically significant enrichment of a subgroup of genes involved in the biological processes located within the neuronal structures, including axons, cell body and somato-dendritic compartment.

**Fig. 7:**
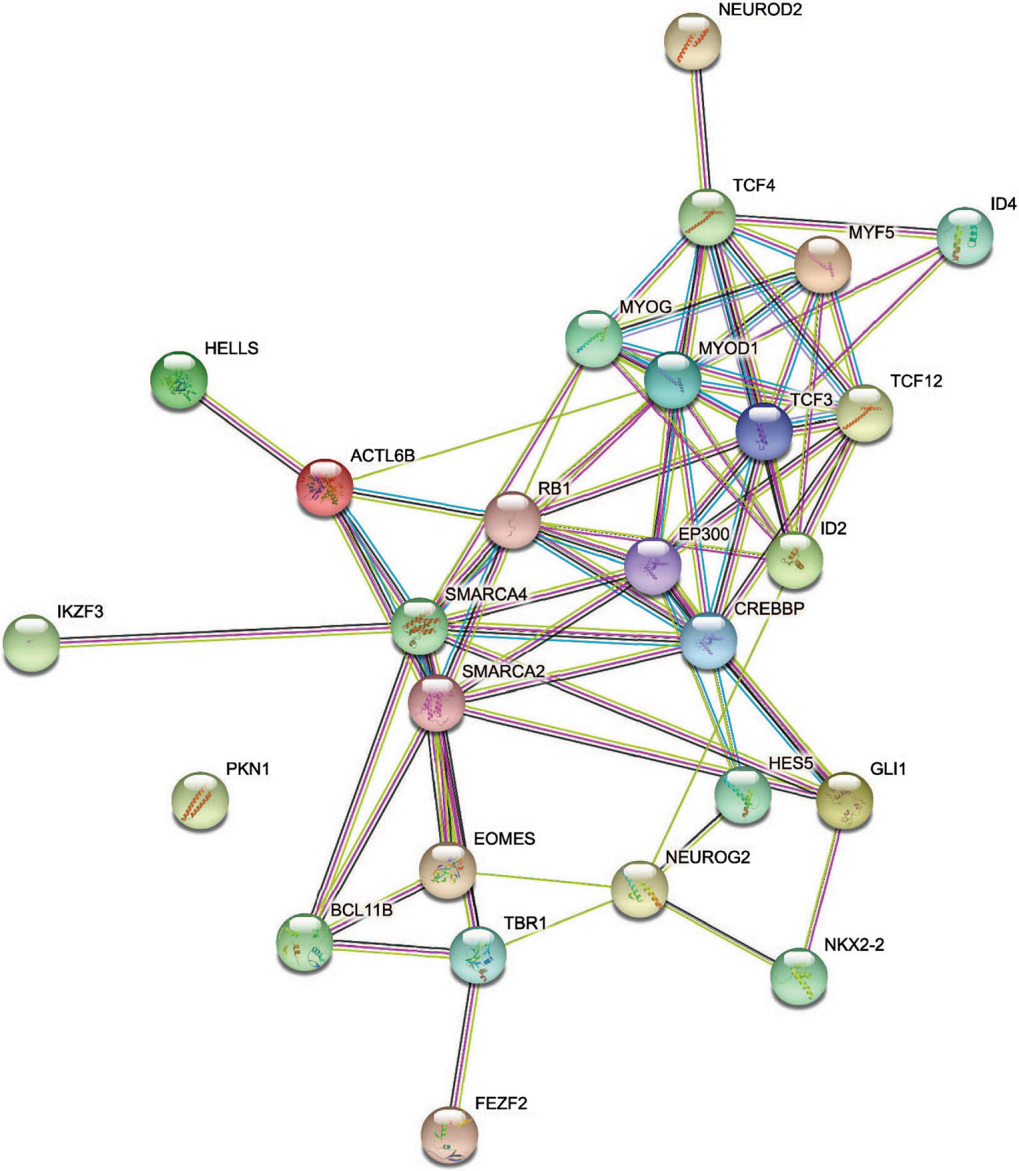
Network showing protein-protein interaction (PPI) among identified target genes

In order to clarify the impact of such genes at a molecular and cellular level, and to better understand which biological function is mainly affected by their dysregulation, we constructed a protein-protein interaction network. In particular, we submitted the target genes to the KEGG pathway and Gene Ontology tools, both implemented in the String database. As shown in Fig. 7, one of the central nodes is *NEUROD2*, which is a transcriptional regulator implicated in neuronal differentiation. *NEUROG2*, *TCF3*, *TCF4* and *TBR1* are also involved in this pathway and likely contribute to its dysregulation.

## Discussion

MiRNAs are a family of short, single-stranded 21–22 nucleotide sncRNAs that act as gene expression modulators at a post-transcriptional level. They are widely distributed within the nervous system and their potential role in the pathogenesis of neurodegenerative disorders is today an active field of research [22]. Recent studies have highlighted the microRNA regulatory functions and impact on neurodegenerative diseases. MicroRNAs have been described as the main regulator of homeostasis in neurons, and their dysregulation results in pathological conditions in the brain [23]. The most common neurodegenerative diseases are Alzheimer’s disease (AD), Parkinson’s disease (PD), Huntington’s disease (HD) and amyotrophic lateral sclerosis (ALS). miRNA deregulation is emerging as a contributor to neurodegeneration by influencing most of the mechanisms responsible for such diseases. In particular, miR-185 and SEPT5 genes may contribute to Parkinson’s disease pathophysiology [24]. MicroRNA 195 triggers neuroinflammation in Parkinson’s disease in a Rho-associated kinase 1-dependent manner [25]. microRNA-137 inhibits Tau hyperphosphorylation in Alzheimer’s disease and targets the CACNA1C gene in transgenic mice and human neuroblastoma SH-SY5Y cells [26]. MCI is defined as an intermediate state between normal ageing and dementia, including AD. Early identification of MCI subjects who will progress to AD is of crucial importance for inclusion in future trials with pathogenic therapies. The current criteria to diagnose this condition require either a lumbar puncture or an amyloid tracer PET scan, but an increasing interest is emerging towards the identification of new, non-invasive and more accessible biomarkers. In this research, we characterized the miRNA expression profile in blood, serum and CSF samples from MCI-AD patients rigorously diagnosed according to the IWG-2 criteria, in order to investigate about the molecular mechanisms underlying the early stages of AD-related cognitive decline and possibly identify potential disease biomarkers that could be eligible for further studies. In particular, we evaluated the expression profile of four selected miRNAs (hsa-mir-5588-5p, hsa-mir-3658, hsa-mir-567, hsa-mir-3908); among these, only hsa-mir-567 was found to be largely expressed in all examined tissues. Noteworthy, we observed a higher expression of hsa-mir-567 in MCI-AD than AD patients. This may reflect a more relevant role of hsa-mir-567 in the earliest stages of cognitive impairment, whereas the impact of its dysregulation could decrease when the disease, and the consequent neuronal loss, progresses. However, both MCI-AD and AD groups exhibited a significantly higher serum expression than healthy controls group, therefore confirming its likely role in determining cognitive decline.

To explore the putative biological roles of hsa-mir-567, we performed a functional annotation procedure, obtaining a cohort of target genes. This analysis confirmed the effect of hsa-mir-567 on genes mainly involved in the biological processes located within the neuronal cells. Noteworthy, many of these genes play a crucial role in neuronal differentiation and normal brain development. In particular, *NEUROD2* (neurogenic differentiation factor 2) codes for a transcriptional factor involved in the establishment and maturation of thalamo-cortical projections, as well as in development of cerebellar and hippocampal granular neurons [27, 28]. *NEUROG2* (Neurogenin-2) encodes a neural-specific basic helix-loop-helix transcription factor that specifies a neuronal fate on ectodermal cells and is expressed in neural progenitor cells within the developing central and peripheral nervous systems [29, 30]. *TCF3* (transcription factor 3, also known as E2A immunoglobulin enhancer-binding factors E12/E47), *TCF4* (transcription factor 4, also known as immunoglobulin transcription factor 2) and *TBR1* (T-box, brain, 1) are other transcriptional factors involved in this pathway [31–33]. In summary, the possible role of hsa-mir-567 in the molecular pathogenesis of AD is suggested not only by its increased expression in serum from both MCI-AD and AD groups compared with healthy controls but also by the analysis of putative target genes, which are mainly involved in neuronal differentiation and in brain connection maturation. Moreover, hsa-mir-567 has been identified in CSF as well as in other more accessible samples, including serum and whole blood, thus representing a potential promising biomarker.

Our study has some limitations. The main one is the reduced size of the sample and consequent low statistical power, so our results need to be replicated on larger cohorts. The strengths of our paper include its primary focus on well-characterized MCI-AD patients, rigorously diagnosed with the IWG-2 criteria. To the best of our knowledge, and in agreement with a recent review by Piscopo and colleagues [34], few papers have examined the miRNA signature in this category of subjects, and none used these criteria for the selection of patients to be included.

Among these, miR-92a-3p, miR-181c-5p and miR-210-3p in plasma have been shown as a specific molecular signature, useful as potential biomarker for AD [35]. Mir-206 displayed a strong correlation with cognitive decline and memory deficit in MCI subjects progressing towards AD [17]. However, the IWG-2 criteria for patient selection and further assessment are needed to confirm if change in these miRNA signatures might correlate to AD biomarkers.

Furthermore, dysregulation of specific miRNAs in CSF samples from MCI-AD subjects has only rarely been sought in the past and with inconclusive results [36].

## Conclusion

The finding of miR-567 upregulation represents a starting point for future research aimed at exploring the roles of hsa-mir-567 in the pathogenesis of AD-related cognitive decline since its earliest stages. Moreover, such results will be confirmed in a larger cohort of patients and their possible use as diagnostic tools or even as biomarkers of MCI progression.
